# Promoting Psychological Resilience Against Academic Burnout: A JD-R Framework Analysis of Self-Compassion as a Mental Health Resource for Diverse Student Populations

**DOI:** 10.3390/healthcare14111585

**Published:** 2026-06-04

**Authors:** Hana Jo, Cho-Eun Yu, Yuna Kim, Sejin Lee, Soo-Jung An

**Affiliations:** 1 Graduate School of Clinical Counseling Psychology, CHA University, Seongnam-si 13488, Republic of Korea; 2 Department of Clinical Psychology, Chungang University, Seoul 06974, Republic of Korea; 3 Graduate School of Counseling Psychology, The Catholic University of Korea, Seoul 06591, Republic of Korea; 4 Department of Counseling Psychology, Myongji University, Seoul 03674, Republic of Korea

**Keywords:** academic burnout, self-compassion, mental wellbeing, social comparison, social support, adult learners

## Abstract

Background/Objectives: Mental health challenges among university students represent a growing public health concern, underscoring the need to understand how psychological resources shape students’ responses to academic stress. Grounded in the Job Demands–Resources (JD-R) framework, this study examined how academic demands (time pressure, college life stress, and social comparison) and a key resource (social support) relate to academic burnout and whether these relationships are moderated by self-compassion. Methods: Participants were 323 Korean undergraduates, including 187 traditional students and 136 adult learners. A moderated moderation analysis was conducted using PROCESS Model 3 to examine the interactive effects of learner type, self-compassion, and academic demands/resources on academic burnout. Results: College life stress and social comparison showed robust positive associations with academic burnout, whereas time pressure showed a weaker association. Social support was not directly associated with lower burnout; instead, its protective role emerged only at moderate to high levels of self-compassion. Self-compassion also demonstrated a differentiated moderating effect across learner groups. Among traditional students, higher self-compassion weakened the association between social comparison and burnout. Among adult learners, the relationship between social support and burnout varied according to levels of self-compassion. Conclusions: Self-compassion emerged as a developmentally relevant personal resource associated with differences in how academic demands and resources relate to burnout. These findings suggest that the effectiveness of external resources may depend on individuals’ capacity to interpret and utilize them, highlighting the importance of self-compassion in both theoretical models of academic burnout and targeted mental health interventions in higher education.

## 1. Introduction

In higher education, academic burnout has become a central concern, not only because of its high prevalence among students, but because of its enduring consequences for academic engagement, psychological well-being, and persistence in learning. Importantly, burnout is increasingly understood not merely as a reaction to excessive workload, but as a process shaped by how academic demands are appraised and regulated in relation to available resources. Despite substantial institutional investment in student support systems, the effectiveness of these resources remains uneven—suggesting that the problem lies not only in the presence of demands or resources, but in how they are psychologically interpreted and utilized.

The Job Demands–Resources (JD-R) framework has emerged as one of the most influential theoretical models for explaining burnout across occupational and educational contexts [1]. According to JD-R theory, burnout develops when demands requiring sustained effort outweigh the resources available to buffer their impact. This framework has been widely applied to university students, identifying academic workload, time pressure, and evaluative stress as key demands, and social support or personal strengths as critical resources [2,3]. However, when extended to increasingly diverse student populations, JD-R theory carries an implicit assumption that warrants closer scrutiny: namely, that resources are uniformly protective across individuals and contexts.

This assumption constitutes a critical blind spot in contemporary higher education. Universities now serve developmentally heterogeneous learners, including traditional students in emerging adulthood and adult learners who return to education after extended life and work experience. These groups differ not only in age but in motivational orientation, identity development, emotional regulation capacity, and exposure to chronic role strain. Yet, most JD-R-based research treats learner populations as psychologically homogeneous, offering limited explanation for why the same academic environment or support resource may protect some students while leaving others vulnerable. Although prior extensions of the JD-R model have incorporated personal resources, such as optimism or self-efficacy, these resources are often conceptualized as static buffers rather than as mechanisms that actively shape the meaning and impact of stressors [4].

To address this gap, the present study advances a developmentally contextualized perspective on JD-R theory by focusing on how personal resources may be associated with the psychological activation of demands and resources. We propose that personal resources do not merely attenuate stress intensity but function as interpretive mechanisms that shape whether demands are experienced as manageable challenges or as emotionally depleting threats. From this perspective, the effectiveness of external resources—such as social support—depends on an individual’s internal readiness to receive, integrate, and utilize such support. Without this interpretive capacity, resources may fail to exert their intended protective effects.

Self-compassion represents a particularly relevant personal resource for examining this process. Defined as a compassionate and nonjudgmental stance toward oneself during moments of difficulty or failure, self-compassion directly targets core psychological processes implicated in burnout, including self-criticism, evaluative threat, and performance-based self-worth [4,5]. Unlike other personal resources such as self-efficacy, which primarily concern perceived behavioral competence, or resilience, which focuses on recovery from adversity, self-compassion operates at the level of cognitive-emotional appraisal, shaping how individuals interpret academic setbacks and social evaluation. In contexts characterized by frequent social comparison, self-compassion may be especially relevant because it interrupts the self-critical appraisal cycle through which unfavorable comparisons are translated into emotional exhaustion—a mechanism less directly addressed by constructs such as self-efficacy or resilience, which primarily emphasize competence beliefs or recovery from adversity rather than the reappraisal of self-evaluative threat [5,6,7]. This makes self-compassion a theoretically grounded candidate for examining how personal resources may moderate burnout risk across student populations with distinct developmental and contextual characteristics [8,9,10]. This theoretical framing is especially salient when considering learner type. Traditional college students, typically in emerging adulthood, are often highly sensitive to peer comparison and social evaluation as they navigate identity formation and academic self-definition. In contrast, adult learners tend to hold more stable self-concepts and self-referenced goals, but face sustained demands arising from role conflict across work, family, and study domains. These developmental differences suggest that both the nature of academic demands and the functionality of personal resources vary systematically by learner type. Yet, empirical research rarely examines how personal resources such as self-compassion operate differently across these developmental contexts within a unified theoretical model.

Against this backdrop, the present study applies a moderated moderation framework grounded in JD-R theory to examine how key academic demands (time pressure, college life stress, and social comparison tendency) and an academic resource (social support) relate to academic burnout, and whether self-compassion alters these relationships differently for traditional students and adult learners. By foregrounding the blind spot of uniform resource effectiveness in JD-R theory, this study seeks to clarify when and for whom resources may function as protective factors, and to position self-compassion as a developmentally sensitive interpretive mechanism within contemporary models of academic burnout.

### 1.1. Conceptualizing Academic Burnout and JD-R Theory

To clarify this contribution, we first outline academic burnout and the JD-R framework, and then specify how a developmental lens reveals when demands and resources may function as psychologically consequential. Although burnout has been conceptualized in diverse ways across occupational, clinical, and educational research, recent reviews have highlighted substantial heterogeneity in its definitions and measurement approaches, with more than 140 definitions identified across different contexts [11]. In the present study, academic burnout is conceptualized based on the multidimensional framework proposed by Schaufeli et al. [12], which defines burnout as emotional exhaustion, cynicism toward academic activities, and reduced academic efficacy. This framework was adopted because it was developed specifically for educational settings, has been widely validated across university student populations and captures distress specifically associated with prolonged academic demands rather than general psychological distress or unrelated life stressors. Although academic burnout is not a formal clinical diagnosis, it has been extensively studied as a theoretically grounded and psychometrically measurable psychological construct. Consistent with contemporary burnout research, the present study does not seek to diagnose burnout at the individual level but rather examines variation in self-reported burnout experiences using a validated educational measure. The scientific basis of the study therefore rests on the integration of a well-established theoretical framework (JD-R theory) and a psychometrically validated operationalization of academic burnout in educational contexts.

Importantly, academic burnout as operationalized in this study is conceptually distinct from burnout arising from occupational, family, financial, or other life domains. The construct is anchored to academically specific experiences—including exhaustion from studying, cynicism toward coursework, and diminished academic efficacy—and was assessed using a measure explicitly framed in relation to academic activities. Accordingly, although students may experience stress across multiple life domains, the present study focuses specifically on burnout associated with academic demands and educational functioning.

Burnout, originally conceptualized by Freudenberger [13] as a state of emotional and physical depletion resulting from prolonged task engagement, has been widely studied in occupational contexts. However, research has increasingly extended to academic settings, recognizing similarities between work-related and academic demands [3,14,15]. Academic burnout has been consistently associated with psychological distress, academic maladjustment, absenteeism, and dropout [3,16].

The Job Demands–Resources (JD-R) theory provides a robust framework for understanding burnout across various contexts, including education [1]. According to JD-R theory, burnout results from an imbalance between excessive demands and insufficient resources. Academic demands—such as time pressure, workload, and evaluative stress—require sustained mental and emotional effort, whereas academic resources—such as social support from peers or instructors—help buffer stress and foster engagement. Moreover, personal resources, particularly self-compassion, have emerged as critical variables that shape how students appraise and respond to academic challenges [2,17].

### 1.2. Key Academic Demands and Resources

Among various academic demands, time pressure is a salient stressor for students who must juggle multiple tasks under tight deadlines. Chronic experiences of time scarcity have been shown to increase exhaustion and academic disengagement [18,19,20]. College life stress, another multifaceted demand, encompasses not only academic pressure but also financial difficulties, interpersonal conflict, and uncertainty about career development [21,22]. Accumulated stress across life domains can undermine resilience and increase susceptibility to burnout.

Social comparison tendency, defined as the propensity to evaluate oneself in relation to others [23], is particularly relevant in achievement-driven educational contexts. Research indicates that Korean students show stronger tendencies toward social comparison than their Western counterparts [24], and those with high comparison tendencies are more likely to experience academic burnout through cycles of self-criticism and diminished self-worth [25,26].

In contrast, social support—encompassing emotional, informational, and instrumental assistance from family, friends, and faculty—has been recognized as a potential protective resource that mitigates the negative effects of stress [27,28]. However, the effectiveness of support may depend on how it is perceived and internalized, suggesting that personal attributes such as self-compassion play a pivotal role in shaping its impact.

### 1.3. Self-Compassion as a Protective Psychological Resource

Self-compassion refers to the ability to treat oneself with kindness and understanding during moments of failure, pain, or inadequacy [17]. It includes three components—self-kindness, common humanity, and mindfulness—and is associated with greater emotional regulation, psychological well-being, and resilience [29,30,31]. In academic contexts, self-compassion has been linked to lower levels of academic burnout, enhanced coping abilities, and reduced negative affect [32,33]. Students with higher self-compassion tend to appraise academic setbacks as part of the learning process rather than personal failure, thereby reducing the emotional toll of academic demands and enhancing the buffer provided by social support.

### 1.4. Expanding the Lens: The Role of Learner Type

Previous studies have reported generational differences in burnout experiences [34,35,36]. Although traditional students and adult learners may differ in generational cohort membership, the present study was not designed as a comparison of generations per se. Rather, learner type was conceptualized as reflecting differences in developmental stage, role responsibilities, and educational context. The inclusion of both groups was intended to examine whether the relationships among academic demands, resources, self-compassion, and burnout vary across learner populations facing distinct developmental and contextual challenges.

While most research on academic burnout has focused on traditional college students, the landscape of higher education is shifting to include a growing population of adult learners—individuals who re-enter formal education after a period of workforce participation [37]. These learners often balance studies with employment, family responsibilities, and community obligations, and thus face unique academic demands, including role conflict and limited time availability [38,39].

The psychological resources and stress responses of adult learners may differ significantly from those of traditional students. For example, adult learners are generally less influenced by peer comparison and more driven by intrinsic goals and personal growth [40]. They may also benefit more from emotional and instrumental support when self-compassion is sufficiently developed. In contrast, traditional students are developmentally more sensitive to social evaluation, competition, and fear of failure, rendering them more susceptible to social comparison and academic burnout [23,25].

Recent national efforts—such as Korea’s expansion of degree-granting programs tailored to adult learners—reflect global trends in lifelong education, aiming to accommodate increasingly diverse student populations [41]. Given these demographic and educational shifts, it is imperative to investigate how academic burnout and protective factors manifest differently across learner types, in order to inform tailored mental health support and prevention strategies for increasingly diverse university populations.

The cultural context of Korean higher education further underscores the relevance of examining academic burnout in this population. Korea consistently ranks among the top-performing countries in the Programme for International Student Assessment (PISA), reflecting a strong national emphasis on academic achievement and performance-oriented education. However, OECD reports [42] also highlight that Korean students experience exceptionally high levels of academic pressure, long study hours, and competitive learning environments compared to other member countries. While Korean students tend to report relatively high levels of school belonging, these academic demands have been identified as potential risk factors for student well-being, particularly in relation to physical and psychological health. Accordingly, improving student well-being in this context requires not only academic achievement but also the creation of learning environments that reduce fear of failure and support psychological adjustment. These cultural and educational characteristics may intensify social comparison processes and increase vulnerability to academic burnout, thereby highlighting the importance of examining protective psychological resources within this context.

### 1.5. Present Study

Drawing on the JD-R framework, the present study adopts a moderated moderation model to identify how internal psychological resources (self-compassion) and environmental resources (social support) were associated with lower burnout in the context of specific academic demands. By examining whether these protective mechanisms vary by learner type, this study aims to provide empirical evidence for designing targeted mental health interventions that foster resilience and may help reduce burnout risk across diverse student populations. Importantly, it seeks to identify self-compassion as a potentially modifiable resource that may inform mental health promotion and burnout prevention strategies in higher education. Thus, this study aims to contribute to psychological theory by clarifying how personal resources function as context-sensitive mechanisms within the JD-R framework.

### 1.6. Research Questions

1. Are there differences in academic demands and resources between traditional college students and adult learners?
2. Do academic demands (time pressure, college life stress, social comparison tendency) predict academic burnout?
3. Does self-compassion moderate the relationship between academic demands/resources and academic burnout?
4. Does the moderating effect of self-compassion vary by learner type (traditional vs. adult learners)?

## 2. Method

### 2.1. Participants and Procedure

This study recruited undergraduate students enrolled in traditional and adult learning programs from universities across South Korea. Traditional students were defined as those who entered college immediately after high school, while adult learners were defined as individuals who re-entered formal education after a period of work or life experience. Participants were recruited via university online communities and survey platforms. A total of 354 responses were collected, with 31 cases removed due to duplicate participation or insufficient data. The final sample comprised 323 students, including 187 traditional students (57.8%) and 136 adult learners (42.2%).

Demographic characteristics are summarized in Table 1. Traditional students were predominantly female (72.2%), with the majority in their third (30%) or second (28.3%) year of study. Most were in their twenties (93%). In contrast, adult learners were also mostly female (78.7%) but showed a more diverse age distribution, including those in their 30s (19.9%), 40s (5.1%), 50s (8.1%), and over 60 (2.2%). Adult learners were more likely to be employed in full-time positions (63.2%) compared to traditional students, who were mostly studying full-time or engaged in part-time work. Because gender composition may influence psychological outcomes, gender distribution between traditional and adult learners was examined using a chi-square test. No significant difference in gender distribution was observed between the two groups, χ^2^(1) = 1.76, *p* = 0.184. This study was conducted in accordance with the ethical standards of the institutional research committee and the 1964 Helsinki Declaration and its later amendments. Formal ethical approval was not required, as the study involved anonymous survey data from adult participants, posed minimal risk, and did not include sensitive personal information. All participants provided informed consent prior to participation.

### 2.2. Measures

All variables were measured using validated Korean versions of established instruments, with items rated on Likert scales unless otherwise noted.

### 2.3. Academic Demands

#### 2.3.1. Time Pressure

Time pressure was assessed using the 9-item Korean version of the Time Pressure Scale (TPS) [43], adapted by [44]. Responses were rated on a 4-point scale (1 = strongly disagree to 4 = strongly agree), with higher scores indicating greater perceived time constraints. Internal consistency was excellent (Cronbach’s α = 0.90).

#### 2.3.2. College Life Stress

College life stress was measured using a 15-item subset of the Revised Life Stress Scale for College Students (RLSS-CS) [45]. This subset focused on two subdomains relevant to both learner groups: academic stress (7 items) and career-related stress (8 items). Items were rated on a 5-point scale (1 = not at all to 5 = very frequently). Internal consistency was high (α = 0.95 overall; 0.90 for academic stress; 0.92 for career stress).

#### 2.3.3. Social Comparison Orientation

Social comparison orientation was assessed using the Iowa–Netherlands Comparison Orientation Measure (INCOM) developed by Gibbons and Buunk [46] and translated and validated for Korean populations by Choi [47]. The INCOM is a widely used measure of individuals’ tendency to compare themselves with others in social contexts and has demonstrated satisfactory psychometric properties across diverse samples [46]. The scale consists of 11 items, including six items assessing comparison of abilities (e.g., “I often compare my behavior with that of people close to me, such as family members or friends”) and five items assessing comparison of opinions (e.g., “I often like to talk with others to learn about their experiences and opinions”). Responses were rated on a 5-point Likert scale ranging from 1 (strongly disagree) to 5 (strongly agree), with Items 5 and 11 reverse scored. Higher scores indicate a stronger orientation toward social comparison. Reference [46] reported satisfactory internal consistency for the original scale, and [47] reported a Cronbach’s α of 0.83 for the Korean version. In the present study, Cronbach’s α was 0.91 for the total scale, 0.87 for the ability comparison subscale, and 0.81 for the opinion comparison subscale.

### 2.4. Academic Resources

#### Perceived Social Support

Social support was measured using the 25-item Perceived Social Support through Others Scale (PSO) [48], which has been validated for use with Korean university students [49]. It includes four subscales: emotional, informational, instrumental, and evaluative support. Items were rated on a 5-point scale (1 = not at all to 5 = very much). Internal consistency was excellent (α = 0.96).

### 2.5. Personal Resource

#### Self-Compassion

Self-compassion was measured using the Korean version of the Self-Compassion Scale (K-SCS) [17], validated by [50]. The scale includes 26 items across six subscales (e.g., self-kindness, self-judgment, mindfulness). Responses were rated on a 5-point scale (1 = almost never to 5 = almost always). Internal consistency for the total scale was α = 0.90.

### 2.6. Academic Burnout

Academic burnout was measured using the Korean version of the Maslach Burnout Inventory–Student Survey (MBI-SS) [12], adapted by [51]. The scale includes 15 items covering emotional exhaustion (5 items), cynicism (4 items), and reduced academic efficacy (6 items, reverse-scored). Items were rated on a 5-point scale (1 = never to 5 = always). Internal consistency was strong across all subscales (α = 0.95 total; 0.89 for exhaustion; 0.91 for cynicism; 0.91 for inefficacy).

### 2.7. Analytical Strategy

Data were analyzed using IBM SPSS Statistics 28.0 (IBM Corp., Armonk, NY, USA) and PROCESS Macro version 3.5 (Andrew F. Hayes, Columbus, OH, USA) [52]. Prior to hypothesis testing, descriptive statistics, bivariate correlations, and independent samples *t*-tests were conducted to compare academic demands and resources across learner types. Normality assumptions were verified via Kolmogorov–Smirnov and Shapiro–Wilk tests, and multicollinearity was checked using Variance Inflation Factor (VIF), with values below 10 considered acceptable.

To examine the main and interactive effects of academic demands, resources, and self-compassion on academic burnout, a moderated moderation model (Model 3 in PROCESS) was applied. The following regression model was estimated:

Y = *b*_0_ + *b*_1_*X* + *b*_2_*W* + *b*_3_*Z* + *b*_4_*XW* + *b*_5_*XZ* + *b*_6_*WZ* + *b*_7_*XWZ* + *e*

where:

- Y = academic burnout;
- X = academic demand/resource variable;
- W = self-compassion;
- Z = learner type (dummy-coded);
- XW, XZ, WZ, XWZ = two-way and three-way interaction terms.

Five separate models were tested to examine the role of each academic demand/resource variable (i.e., time pressure, college life stress, social comparison, and social support).

The model tests whether the moderating effect of self-compassion (W) on the X–Y relationship is itself moderated by learner type (Z). Significance was assessed using 5000 bootstrapped samples to compute bias-corrected 95% confidence intervals.

**Figure 1:**
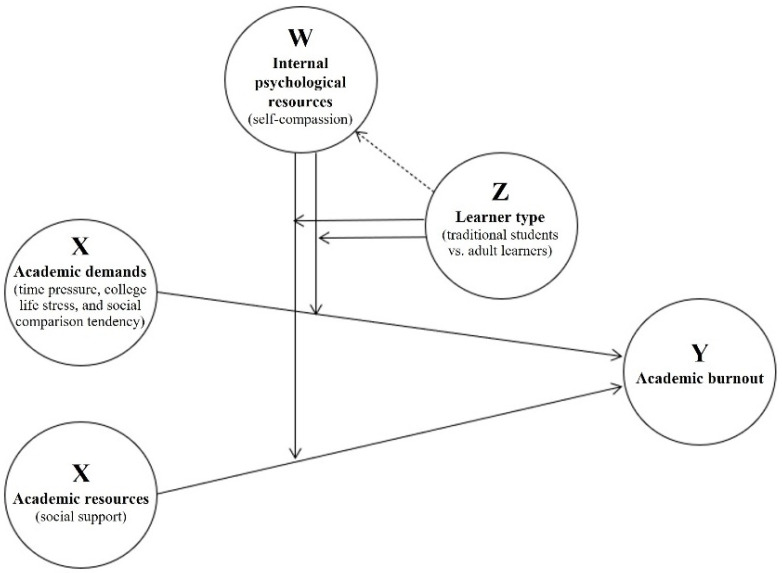
Conceptual model of moderated moderation.

Significant three-way interactions (X × W × Z) were further probed using the Johnson-Neyman technique to identify regions of significance and by conducting simple slopes analyses at ±1 SD of self-compassion within each learner group. As illustrated in Figure 1, the conceptual model represents the moderated moderation structure linking academic demands and resources to academic burnout.

Model comparisons using Akaike Information Criterion (AIC) and Bayesian Information Criterion (BIC) were also performed to assess the fit of the moderated moderation model relative to simpler alternatives.

### 2.8. Ethics Statement

The study involved an anonymous and voluntary online survey of adult university students. Participants were informed about the purpose of the study and provided informed consent before participation. No personally identifiable information or sensitive personal information was collected. During the revision process, the authors consulted the institutional IRB office regarding the ethical context of the study. The IRB office indicated that a definitive retrospective determination regarding the conduct of the previously completed study could not be made; however, the study did not collect personally identifiable or sensitive personal information and appeared to involve minimal risk to participants. All procedures were conducted in accordance with the ethical principles of the Declaration of Helsinki and applicable institutional guidelines at the time of data collection.

## 3. Results

### 3.1. Descriptive Statistics and Correlation

Prior to hypothesis testing, assumptions for regression analysis were examined. The skewness and kurtosis values for all variables were within acceptable ranges (absolute skewness < 2, kurtosis < 4) [53], indicating normality. Multicollinearity was not a concern, as all variance inflation factor (VIF) values ranged from 1.199 to 2.602 [52], well below the critical threshold of 10. The independence of residuals was supported by Durbin–Watson statistics between 1.871 and 1.925, satisfying the assumption of residual independence. Descriptive statistics and correlations among the major variables for each group are presented in Table 2.

### 3.2. Group Differences in Key Variables

Independent samples *t*-tests were conducted to examine differences between traditional college students and adult learners. As shown in Table 3, traditional students reported significantly higher levels of college life stress, social comparison tendency, and academic burnout compared to adult learners. No significant differences were found in time pressure, self-compassion, or social support.

### 3.3. Moderated Moderation Analyses

Four separate moderated moderation analyses were conducted using PROCESS Macro Model 3 [52], one for each predictor (time pressure, college life stress, social comparison tendency, and social support). The outcome variable was academic burnout; self-compassion was the first moderator, and learner type (traditional vs. adult) was the second moderator. Regression results are summarized in Table 4.

*Time Pressure:* Time pressure significantly predicted academic burnout (B = 0.9670, *p* < 0.001), and self-compassion was significantly associated with a buffering pattern (B = −0.4589, *p* < 0.001). Learner type (traditional vs. adult) also significantly moderated burnout levels (B = −0.2980, *p* < 0.001). However, the three-way interaction between time pressure, self-compassion, and learner type was not significant (B = 0.0743, *p* = 0.172), suggesting that the moderating role of self-compassion did not vary by learner type in this context.

*College Stress:* College life stress significantly predicted academic burnout (B = 0.7386, *p* < 0.001). Self-compassion moderated this relationship (B = −0.2690, *p* = 0.0013), and learner type had a significant moderating effect (B = −0.0653, *p* = 0.0432). Importantly, the three-way interaction among college life stress, self-compassion, and learner type was significant (B = −0.0798, *p* = 0.0457), indicating that the buffering effect of self-compassion differed between traditional students and adult learners.

*Social Comparison:* Social comparison tendency was positively associated with academic burnout (B = 0.4316, *p* < 0.001). Self-compassion had a strong buffering effect (B = −0.6859, *p* < 0.001), and learner type also moderated the relationship (B = −0.2097, *p* < 0.001). The three-way interaction was significant (B = 0.1672, *p* = 0.0021), suggesting that the association between self-compassion and burnout protection against social comparison-related burnout differed by learner type.

*Social Support:* Social support alone did not significantly predict academic burnout (B = 0.1557, *p* = 0.3186). However, its interaction with self-compassion was significant (B = −0.3434, *p* = 0.0057), and the three-way interaction with learner type was also significant (B = −0.1993, *p* = 0.0050), suggesting that the effect of self-compassion on the social support-burnout link varied across learner types.

### 3.4. Model Fit

The model fit was evaluated by comparing Akaike Information Criterion (AIC) [54], and Bayesian Information Criterion (BIC) [55] values between simple moderation models and moderated moderation models. As shown in Table 5, across all variables (time pressure, college life stress, social comparison tendency, social support), moderated moderation models exhibited lower AIC and BIC values, indicating superior model fit.

### 3.5. Conditional Effects and Simple Slopes

**Figure 2:**
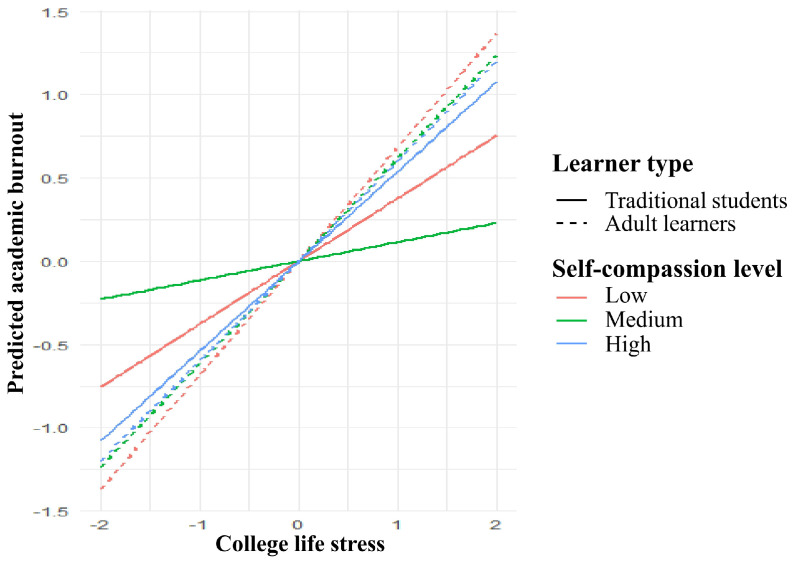
Interaction plot for college life stress × self-compassion × learner type.

**Figure 3:**
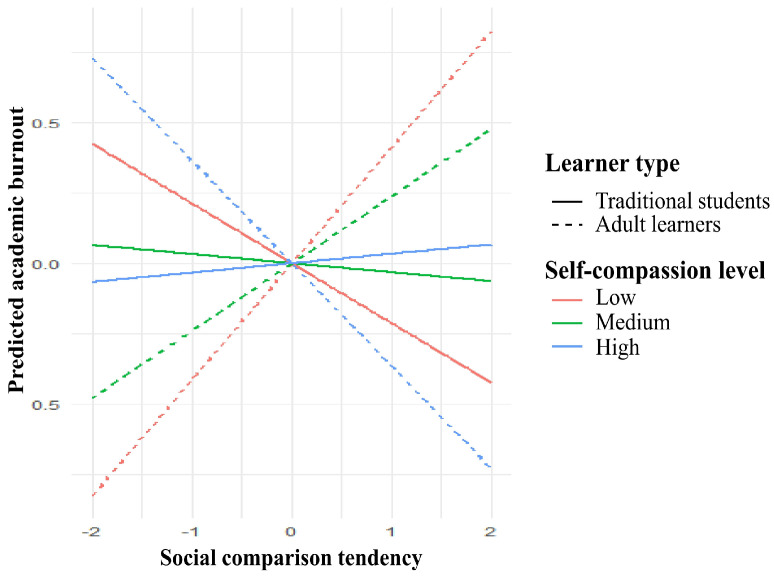
Interaction plot for social comparison × self-compassion × learner type.

**Figure 4:**
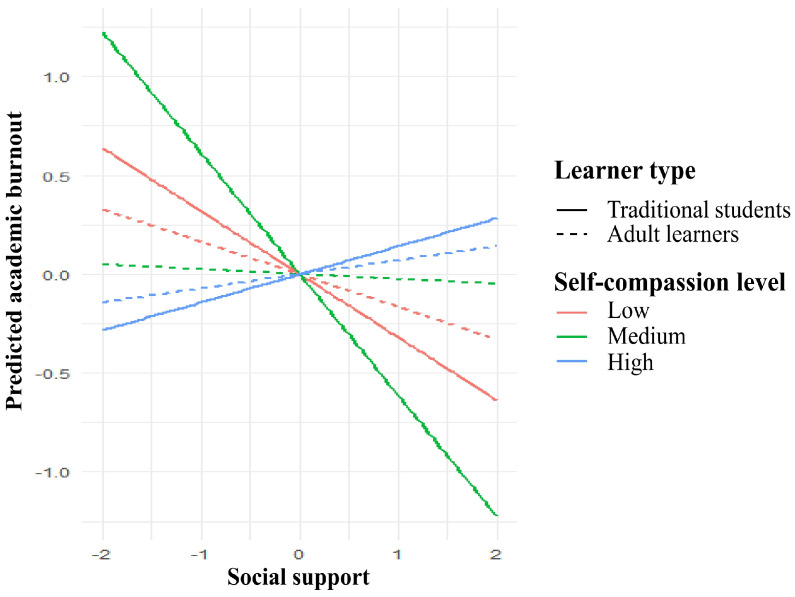
Interaction plot for social support × self-compassion × learner type.

Johnson-Neyman [56] analyses and simple slope tests further illustrated how the effects of academic demands/resources on burnout varied by levels of self-compassion and learner type. For example, among adult learners, self-compassion significantly buffered the effect of college life stress and social support on academic burnout, especially at moderate to high levels of self-compassion. For traditional students, self-compassion was particularly protective in contexts of high social comparison. The conditional effects results are presented in Table 6, and interaction plots are provided in Figure 2, Figure 3 and Figure 4.

## 4. Discussion

The present study contributes to the public health and psychological literature on stress, coping, and burnout processes in higher education by identifying when and for whom psychological resources may function as protective against academic burnout. Rather than merely documenting the effects of academic demands and resources on burnout, the present study advances the Job Demands–Resources (JD-R) framework by clarifying when and for whom resources may function as psychologically protective. In particular, self-compassion emerged not as a general-purpose buffer, but as a developmentally contingent interpretive mechanism that shapes how demands are appraised and how resources are psychologically activated.

By applying a moderated moderation approach, findings suggest that the same academic environment may be associated with qualitatively different burnout processes depending on learner type. Traditional college students and adult learners were not simply exposed to different levels of stress; they differed in how evaluative demands, structural pressures, and social resources were internalized and regulated. These findings expose a critical blind spot in conventional applications of JD-R theory in educational contexts—namely, the assumption that resources function equivalently across developmental stages. Addressing this blind spot allows for a more precise understanding of burnout as a dynamic, context-sensitive process rather than a uniform response to academic overload, and highlights self-compassion as a key target for psychological processes and burnout prevention in diverse university populations.

### 4.1. Developmental Patterns in Burnout and Stress Perception

Traditional students reported higher burnout, college life stress, and social comparison than adult learners, consistent with developmental differences in how academic stress is appraised and regulated. Emerging adults, who are often navigating identity exploration and peer-based evaluation, may be especially reactive to comparative and relational stressors [32]. Adult learners, by contrast, tend to draw on clearer goal structures and more stable self-concepts, which can support emotion regulation and reduce burnout risk [37]. These patterns underscore the need for developmentally attuned prevention approaches rather than “one-size-fits-all” support.

### 4.2. Reconceptualizing Demands: Social Comparison as Psychological Load

All demands were associated with burnout, but social comparison showed the strongest link. Unlike structural stressors (e.g., time pressure), social comparison reflects an internalized evaluative load—fuelled by perceived inadequacy and fear of negative judgment—that is likely amplified during identity formation. This supports treating social comparison as a psychologically activated demand within the JD-R framework [57], and as a central target for intervention when burnout is driven by self-evaluative threat.

### 4.3. When Support Is Not Enough: The Role of Internal Readiness

Social support did not show a direct association with burnout; its protective effect emerged when self-compassion was moderate to high. This pattern suggests that external resources may be beneficial only when learners can metabolize them without self-criticism. Adult learners may experience support as genuine affirmation, whereas traditional students may interpret similar messages through a performance-contingent lens [58]. In this sense, self-compassion functions as a psychological “gatekeeper” that shapes whether support feels nurturing or evaluative. This interpretive process may be particularly relevant within the Korean educational context, where supportive interactions are often embedded within broader expectations regarding achievement, responsibility, and reciprocity [23]. Consequently, the effectiveness of social support may depend not only on its availability but also on how it is perceived and internalized by recipients [24]. Students with greater self-compassion may be better positioned to receive support as genuinely affirming, whereas those lower in self-compassion may experience identical support as a reminder of unmet standards [4]—suggesting that cultural context shapes not only the form that support takes, but the psychological conditions under which it becomes protective.

### 4.4. Self-Compassion as a Developmentally Sensitive Regulator

Across analyses, self-compassion operated as a key personal resource that reduced burnout vulnerability by softening self-criticism and supporting emotion regulation [6,59]. Its buffering effect was most evident for social comparison, suggesting that self-compassion is particularly relevant for internally generated, self-evaluative demands [9]. By contrast, more externally imposed stressors (e.g., time pressure) appeared less amenable to reappraisal, highlighting that “what self-compassion can buffer” depends on demand characteristics. Notably, adult learners showed a broader pattern in which self-compassion also shaped the impact of college life stress and the meaning of support [38], consistent with greater role complexity and reflective capacity. Overall, these findings position self-compassion not merely as an insulator but as an interpreter that alters demand appraisal and resource usability in developmentally specific ways.

### 4.5. Moderated Moderation: Developmentally Contextualized Resource Dynamics

The moderated moderation findings indicate that the buffering role of self-compassion depends on learner type, supporting a developmentally contextualized view of personal resources within the JD-R model [2]. For traditional students, self-compassion primarily attenuated the link between social comparison and burnout, aligning with heightened sensitivity to peer evaluation in emerging adulthood [60,61]. For adult learners, self-compassion was more closely tied to college life stress and the impact of social support, consistent with multi-role demands and more stable self-concepts [38]. Together, these patterns suggest that personal resources are not uniformly protective; their function is contingent on both developmental context and the dominant demand profile.

### 4.6. Theoretical Implications

This study advances the Job Demands–Resources (JD-R) model by demonstrating that academic burnout is fundamentally shaped by developmental context and the psychological interpretation of demands and resources. By comparing traditional and adult learners, the findings highlight that burnout processes vary not only in intensity but in structure, calling for a developmentally contextualized JD-R framework that accounts for life-stage differences in stress appraisal and resource mobilization.

A central theoretical contribution concerns the reconceptualization of academic demands. The robust effect of social comparison—particularly among traditional students—suggests that internally generated, socially evaluative pressures constitute a distinct and potent class of demands. These findings support expanding the JD-R notion of “demand” beyond externally imposed workload to include social-cognitive stressors rooted in identity development and evaluative vulnerability.

Finally, the study refines the role of personal and external resources within the JD-R model. Social support alone was not significantly associated with lower burnout, underscoring that resources are not uniformly protective but are subjectively and interpretively activated. Self-compassion emerged as a developmentally sensitive personal resource associated with differences in demand appraisal and the psychological accessibility of support. Together, these findings position personal resources not as static buffers but as context-dependent activators that shape burnout pathways across developmental stages.

### 4.7. Practical Implications

The present findings offer several practical insights for psychological functioning and burnout prevention in higher education, particularly within developmentally diverse student populations. First, differentiated burnout patterns across learner types highlight the need for tailored interventions. For traditional students, vulnerable to social comparison and self-criticism, self-compassion programs—such as mindfulness-based stress reduction, compassion-focused psychoeducation, and non-competitive peer mentoring—may support emotional safety, normalize academic struggle, and support self-acceptance as a coping resource. Universities may further enhance accessibility by integrating self-compassion and well-being education into first-year orientation programs, general education curricula, or campus-wide mental health initiatives rather than relying solely on voluntary participation in counseling services.

Second, for adult learners, burnout often arises from chronic time pressure, multi-role conflict, and logistical barriers. Practical supports—such as time-management workshops, work–life–study balance coaching, and structural accommodations—may help reduce role strain, and incorporating self-compassion training may help adult learners manage competing demands without self-blame or overextension. To increase feasibility for adult learners balancing work and family responsibilities, self-compassion and well-being interventions may be delivered through flexible formats, including online modules, asynchronous learning resources, and brief skills-based workshops.

Third, the non-uniform impact of social support highlights that its quality and framing may be as important as its availability. Traditional students appear to benefit more from emotionally validating and autonomy-supportive interactions than from achievement-focused encouragement. In contrast, adult learners may benefit from support systems that address the practical constraints associated with balancing work, family, and academic responsibilities. Prior research suggests that financial burden and limited institutional support are common barriers to persistence among adult learners [62,63]. Accordingly, universities may consider a range of supportive resources—including flexible learning opportunities, family-responsive services, academic advising, and, where appropriate, financial assistance—to facilitate sustained academic engagement and effective utilization of available social support [55]. The present findings further suggest that universities should focus not only on increasing the availability of support services but also on fostering conditions in which support is experienced as psychologically safe and affirming. Faculty members, advisors, and student support personnel may therefore benefit from training that emphasizes autonomy-supportive communication, normalization of academic difficulties, and feedback practices that separate academic performance from personal worth.

Fourth, cultivating emotionally supportive campus communities may help buffer burnout. Universities may consider promoting inclusive peer networks, adult learner groups, and intergenerational extracurricular programs, which have been associated with enhanced belonging, relational motivation, and purpose-driven learning, especially for adult learners seeking community and meaning.

Finally, these findings highlight the need for integrated, person-centered support systems tailored to develop mental context. Institutions should address both internal resilience and contextual factors in supporting students’ well-being. In addition, universities should move beyond passive counseling availability toward proactive burnout prevention systems. Routine well-being screening during academically demanding periods, faculty training on burnout recognition, and integrated referral pathways between counseling centers and academic support services may facilitate earlier identification of students at risk. Psychoeducational programs that help students recognize maladaptive perfectionism, excessive social comparison, and early signs of emotional exhaustion may further promote self-monitoring, timely help-seeking, and more self-compassionate responses to academic setbacks. Such approaches may contribute to promoting students’ mental well-being, reducing burnout risk, and supporting sustained engagement and success across diverse higher education populations.

### 4.8. Limitations and Future Directions

Although this study provides a developmentally contextualized account of academic burnout within the JD-R framework, several limitations point to important directions for future research. First, the cross-sectional design precludes causal inference regarding how demands, resources, and self-compassion dynamically interact over time. Longitudinal and experience-sampling approaches would allow future studies to examine how interpretive resources such as self-compassion shape within-person fluctuations in burnout processes.

Second, the exclusive reliance on self-report measures highlights the need for multi-method approaches that capture both subjective appraisal and behavioral regulation. Incorporating qualitative interviews, observational indicators, or peer reports could deepen understanding of how personal resources are enacted in real-world academic contexts, particularly in relation to socially evaluative demands.

Third, although social support was assessed using a multidimensional measure that included emotional, informational, appraisal, and material support, analyses were conducted using an overall support score. Different forms of support may serve distinct functions depending on learners’ developmental and contextual demands. Future research should examine whether specific support types and support sources differentially interact with self-compassion to influence academic burnout across learner populations.

Fourth, academic burnout was examined as a self-reported psychological construct rather than a clinically defined syndrome. Consistent with contemporary burnout research, the present findings therefore reflect individuals’ subjective experiences and appraisals of burnout rather than objectively verified clinical states. Future research employing longitudinal, behavioral, or multi-method assessments may help further strengthen understanding of burnout processes across educational contexts.

Fifth, although no significant difference in gender distribution was observed between traditional and adult learners in the present sample, prior research suggests that burnout experiences and related psychological processes may vary across gender groups. Future research should therefore examine whether the relationships among academic demands, resources, self-compassion, and academic burnout differ as a function of gender.

Finally, learner type in the present study was partially confounded with age and generational cohort membership. Traditional students were predominantly younger adults, whereas adult learners represented a broader age range and may have differed in cohort-related experiences and coping styles. Because prior research has documented generational differences in burnout vulnerability and stress appraisal [29,35], future studies should further disentangle developmental, cohort, and role-related influences on academic burnout.

## 5. Conclusions

This study advances a developmental-contextual account of academic burnout by demonstrating that the effects of academic demands and resources are not uniform, but contingent on learners’ developmental positioning and internal regulatory capacities. By integrating self-compassion and learner type into the Job Demands–Resources (JD-R) framework, the findings highlight a critical blind spot in conventional burnout models—namely, the assumption that resources function equivalently across individuals and contexts.

Across learner types, academic burnout was shaped not only by exposure to demands but also by how those demands were psychologically appraised and regulated. Self-compassion emerged as a key interpretive mechanism associated with lower evaluative pressures and more effective utilization of external support, with its protective role varying systematically by learner type and dominant stressors.

Taken together, these findings underscore the need to move beyond one-size-fits-all approaches to burnout prevention in higher education. Conceptualizing personal resources as developmentally contingent activators rather than static buffers offers a more precise framework for understanding academic burnout in increasingly diverse learning populations and may provide a foundation for psychologically sustainable support systems across the lifespan of educational development. Importantly, these insights contribute to the design of targeted psychological interventions that may support student wellbeing and reduce burnout risk, and support long-term academic engagement across diverse learner groups.

## Figures and Tables

**Table 1:** Demographic characteristics of participants.

Characteristic	Category	Traditional College Students (*n* = 187) *n* (%)	Adult Learners (*n* = 136) *n* (%)
Age	20s	174 (93.0%)	88 (64.7%)
	30s	13 (7.0%)	27 (19.9%)
	40s		7 (5.1%)
	50s		11 (8.1%)
	60s and above		3 (2.2%)
Gender	Female	135 (72.2%)	107 (78.7%)
	Male	52 (27.8%)	29 (21.3%)
Academic Year	1st Year	24 (12.8%)	39 (28.7%)
	2nd Year	53 (28.3%)	17 (12.5%)
	3rd Year	56 (30.0%)	41 (30.1%)
	4th Year	50 (26.8%)	34 (25.0%)
	On leave	4 (2.1%)	5 (3.7%)
University Location	Seoul, Gyeonggi, Incheon	138 (73.8%)	123 (90.5%)
	Daejeon, Gwangju, Daegu	24 (12.8%)	6 (4.4%)
	Ulsan, Busan	14 (7.5%)	3 (2.2%)
	Gangwon, Chungcheong	5 (2.7%)	0 (0.0%)
	Gyeongsang, Jeolla, Jeju	6 (3.2%)	4 (2.9%)
Occupation	Full-time job		86 (63.2%)
	Part-time job	69 (36.9%)	10 (7.4%)
	Studying only	117 (62.6%)	34 (25.0%)
	Other	1 (0.5%)	6 (4.4%)

Note. Gender distribution did not significantly differ between traditional and adult learners, χ^2^(1) = 1.76, *p* = 0.184.

**Table 2:** Descriptive statistics and correlations among study variables by learner group.

Variables	1	2	3	4	5	6	7	8
**Traditional Students**								
1. Time Pressure	1							
2. College Stress	0.873 **	1						
3. Social Comparison	0.432 **	0.418 **	1					
4. Social Support	−0.426 **	−0.496 **	−0.136	1				
5. Academic Burnout	0.832 **	0.860 **	0.414 **	−0.593 **	1			
6. Self-Compassion	−0.692 **	−0.693 **	−0.409 **	0.693 **	−0.805 **	1		
7. Gender	0.103	0.094	−0.102	−0.204 **	0.066	−0.026	1	
8. Employment	−0.515 **	−0.436 **	−0.229 **	0.225 **	−0.473 **	0.299 **	−0.026	1
*M*	2.69	3.11	3.49	3.84	2.80	3.25		
*SD*	0.854	1.111	0.978	0.709	1.10	1.05		
Skewness	−0.308	−0.362	−0.453	−0.608	0.140	0.008		
Kurtosis	−1.20	−1.24	−0.934	−0.291	−1.17	−1.32		
**Adult Learners**								
1. Time Pressure	1							
2. College Stress	0.519 **	1						
3. Social Comparison	0.105	0.013	1					
4. Social Support	−0.134	−0.203 *	0.152	1				
5. Academic Burnout	0.349 **	0.402 **	0.043	−0.504 **	1			
6. Self-Compassion	−0.283 **	−0.198 *	−0.285 **	0.467 **	−0.619 **	1		
7. Gender	−0.001	0.002	−0.052	−0.240 **	0.041	−0.021	1	
8. Employment	−0.237 **	−0.025	−0.014	0.172 *	−0.299 **	0.257 **	−0.154	1
*M*	2.77	2.82	3.20	3.87	2.46	3.34		
*SD*	0.621	0.754	0.700	0.693	0.752	0.722		
Skewness	−0.576	−0.143	0.048	−0.657	−0.019	0.199		
Kurtosis	−0.087	−0.223	−0.639	0.074	−0.887	−0.545		

Note. Across both groups, time pressure and college life stress were significantly and positively associated with academic burnout. Social comparison was positively associated with burnout only among traditional students, whereas this association was not significant among adult learners. In contrast, self-compassion and social support were significantly and negatively associated with academic burnout in both groups. * *p* < 0.05, ** *p* < 0.01.

**Table 3:** Group differences in academic demands, resources, and burnout.

Variables	Traditional Students (*n* = 187)	Adult Learners (*n* = 136)	*t*-Test
	M (SD)	M (SD)	
Time pressure	2.69 (0.85)	2.77 (0.62)	−0.91
College life stress	3.11 (1.11)	2.82 (0.75)	2.76 **
Social comparison	3.49 (0.98)	3.20 (0.75)	3.08 **
Social support	3.84 (0.71)	3.87 (0.69)	−0.34
Academic burnout	2.80 (1.10)	2.45 (0.75)	3.35 ***
Self-compassion	3.25 (1.05)	3.34 (0.72)	−0.93

** *p* < 0.01, *** *p* < 0.001.

**Table 4:** Analysis results of the moderated moderation model.

Variables	Main Effect B (*p*)	Moderation by Self-Compassion B (*p*)	Moderation by Learner Type B (*p*)	Three-Way Interaction B (*p*)
Time Pressure	0.9670 (<0.001)	−0.4589 (<0.001)	−0.2980 (<0.001)	0.0743 (0.172)
College Life Stress	0.7386 (<0.001)	−0.2690 (0.0013)	−0.0653 (0.0432)	−0.0798 (0.0457)
Social Comparison	0.4316 (<0.001)	−0.6859 (<0.001)	−0.2097 (0.0001)	0.1672 (0.0021)
Social Support	0.1557 (0.3186)	−0.3434 (0.0057)	−0.1663 (0.0136)	−0.1993 (0.0050)

**Table 5:** Model fit comparison between simple and moderated moderation models.

Variables	Simple Moderation Model		Moderated Moderation Model	
	AIC	BIC	AIC	BIC
Time Pressure	572.983	575.604	530.111	533.844
College Life Stress	499.840	503.590	490.954	494.694
Social Comparison	640.890	644.639	629.686	633.426
Social Support	639.295	643.044	619.026	622.766

**Table 6:** Conditional effects at different levels of self-compassion and learner type.

Learner Type	Level of Self-Compassion	College Life Stress	Social Comparison	Social Support
Traditional College Students	Low (−0.9273)	0.6741 (<0.001)	0.3871 (0.0001)	−0.1443 (0.1105)
	Medium (0)	0.6109 (<0.001)	0.2219 (0.0002)	−0.0106 (0.9150)
	High (0.9274)	0.5477 (<0.001)	0.0567 (0.2860)	0.1230 (0.3907)
Adult Learners	Low	0.5668 (<0.001)	−0.3423 (0.0166)	−0.1073 (0.3853)
	Medium	0.3555 (<0.001)	−0.1974 (0.0208)	−0.3433 (0.0002)
	High	0.1443 (0.0531)	−0.0526 (0.5906)	−0.5793 (0.0004)

## Data Availability

The datasets generated and/or analyzed during the current study are available from the corresponding author upon reasonable request but are not publicly available due to privacy and ethical restrictions.
